# An integrated resuscitation service, combining a pre - hospital physician response unit with delivery to a dedicated high-volume cardiac arrest centre, optimises survival following cardiac arrest

**DOI:** 10.1186/1757-7241-22-S1-P17

**Published:** 2014-07-07

**Authors:** Ceri Hunter-Dunn

**Affiliations:** 1 London’s Air Ambulance, London E1 1BB, UK

## Purpose & background

Despite recent advances in resuscitation medicine, survival rates to discharge following out-of-hospital cardiac arrest (OHCA) remain poor. Recent data support the implementation of early critical care interventions including therapeutic hypothermia (TH) and immediate percutaneous coronary intervention (PCI). We describe a pre-hospital service where early delivery of critical care interventions and the capacity to transfer these unstable patients directly to a cardiac intervention centre is available through the deployment of a specialist physician response unit (PRU).

## Methods

Prospective data were gathered on all cases of OHCA attended by the PRU, transferred to a single interventional cardiology centre. Data included patient demographics, clinical characteristics, clinical interventions at the scene and at hospital, and clinical outcome.

## Results

Between 14^th^ July 2013 and 19^th^ August 2013, data were obtained on 28 patients, with a mean age of 59 (range 29-85). Twenty-three were male (82%). Presenting rhythms were ventricular fibrillation (VF) 68%, asystole 14%, pulseless electrical activity 11% and ventricular tachycardia (VT) 7%. There were return of spontaneous circulation (ROSC) at scene in 89%. PRU interventions included advanced airway management 90%, rapid sequence induction 71%, early TH 89%, mechanical load distributing band CPR device was used in 82% and mechanical resuscitation during transfer 14%.

Survival data were as follows: Overall survival to cardiac intensive care (CICU) was 56%, with overall survival to discharge 48%. Of those patients (17) who underwent emergency angiography, 76% survived to CICU, with 64% surviving to discharge. Of patients presenting in VF, 62% survived to discharge. Interestingly, 72% of patients who received TH survived to discharge. All patients who underwent successful PCI survived to discharge with good neurological outcomes.

## Conclusions

In selected cases, outcomes following OHCA can be optimized by integrating a physician-led cardiac arrest service enabling urgent delivery of patients to a cardiac arrest centre, with the early application of neuro-protective strategies, circulatory support and interventional cardiology techniques.

